# Supplementary material for: BioConvert: a comprehensive format converter for life sciences
Source: NAR Genom Bioinform. 2023 Aug 21;5(3):lqad074. doi: 10.1093/nargab/lqad074 (PMC10440784; doi:10.1093/nargab/lqad074)
Supplement: lqad074_Supplemental_File [file lqad074_supplemental_file.pdf]

SUPPLEMENTARY

Workflows and parellelisation

Life sciences data, particularly in NGS-related fields, can be quite large, necessitating parallelization of conversion most of the time. **BioConvert**, being a standalone application, can easily be integrated into any framework that utilizes embarrassingly parallel paradigm.

Snakemake example. As an illustration, we provide an example that demonstrates the usage of the Snakemake (1) framework. In this context, it is necessary to specify the input and output files, along with their respective extensions, and the conversion command.

```
import glob

in_ext = "fastq.gz"
out_ext = "fasta"
command = "fastq2fasta"
location = "."

# nothing to change below
samples = glob.glob(f"{location}/*.{in_ext}")
samples = [x.rsplit(".")[0] for x in samples]
rule all:
  input: expand(f"{{dataset}}.{out_ext}",
               dataset=samples)

rule bioconvert:
  input: f"{{dataset}}.{in_ext}"
  output: f"{{dataset}}.{out_ext}"
  run:
    cmd = f"bioconvert {command}"
    cmd += f" {{input}} {{output}}"
    shell(cmd)
```

Figure 1. Example of a Snakemake integration

Nextflow bioconvert pipeline. Similarly, we provide a simple example for the NextFlow (2) framework.

Sequana bioconvert pipeline. Sequana provides NGS pipelines developed in Snakemake (sequana.readthedocs.io) (3). It also provide a pipeline dedicated to **BioConvert** that can be installed as follows:

```
pip install sequana_bioconvert
```

Then, a user that wishes to convert a set of BAM files into SAM files would need to type the following commands without the need to install third-party tools (here samtools) or wonder about Snakemake syntax.

```
    sequana_bioconvert --input-directory data/
       --input-ext "fastq.gz"
       --output-ext "fasta.gz"
       --use-apptainer
       --apptainer-prefix ~/images/
       --command fastq2fasta
       --input-pattern "*"
```

This command downloads the required container automatically and perform the conversion on the input files.

```
nextflow.enable.dsl=2

process bioconvert {
  publishDir "results", mode: 'copy'
  container "quay.io/biocontainers/bioconvert"

  input:
    path infile
    val out_ext
    val command

  output:
    path "${infile.simpleName}.${out_ext}"

  script:
    """
    bioconvert ${command} \
               ${infile} \
               ${infile.simpleName}.${out_ext}
    """
}

workflow {
  in_ext = "fastq.gz"
  out_ext = "fasta"
  command = "fastq2fasta"
  inpath = "."
  infiles= channel.fromPath(inpath+"/*."+in_ext)
  bioconvert(infiles, out_ext, command)
}
```

Figure 2. Example of a Nextflow integration

REFERENCES

1. Mölder, F., Jablonski, K., Letcher, B., Hall, M., Tomkins-Tinch, C., Sochat, V., Forster, J., Lee, S., Twardziok, S., Kanitz, A., Wilm, A., Holtgrewe, M., Rahmann, S., Nahnsen, S., and Köster, J. (2021) Sustainable data analysis with Snakemake. F1000Research, **10**(33).
2. Di Tommaso, P. e. a. (2017) Nextflow enables reproducible computational workflows.. Nature Biotechnology, **35**.
3. Cokelaer, T., Desvillechabrol, D., Legendre, R., and Cardon, M. (2017) 'Sequana': a set of Snakemake NGS pipelines. Journal of Open Source Software, **2**(16), 352.
